# Ultrasound-Guided Selective Bronchial Intubation: A Feasibility Study in Pediatric Animal Model

**DOI:** 10.3389/fmed.2022.869771

**Published:** 2022-06-15

**Authors:** Sara Hora Gomes, Alice Miranda, José Miguel Pêgo, Patrício S. Costa, Jorge Correia-Pinto

**Affiliations:** ^1^Life and Health Sciences Research Institute (ICVS), School of Medicine, University of Minho, Braga, Portugal; ^2^ICVS/3B’s-PT Government Associate Laboratory, Braga, Portugal; ^3^Department of Pediatric Surgery, Hospital de Braga, Braga, Portugal

**Keywords:** ultrasound-guided, selective, bronchial intubation, bronchial exclusion, pediatric animal model

## Abstract

**Objective:**

Selective one-lung ventilation used to optimize neonatal and pediatric surgical conditions is always a demanding task for anesthesiologists, especially during minimally invasive thoracoscopic surgery. This study aims to introduce an ultrasound-guided bronchial intubation and exclusion technique in a pediatric animal model.

**Methods:**

Seven rabbits were anesthetized and airway ultrasound acquisitions were done.

**Results:**

Tracheal tube progression along the trachea to the right bronchus and positioning of the bronchial blocker in the left bronchus were successfully done with consistent ultrasound identification of relevant anatomical structures.

**Conclusion:**

The study provided a new application of ultrasound in airway management. More advanced experimental studies are needed since this technique has the potential for translation to pediatric anesthesia.

## Introduction

One lung ventilation in neonates and very small children is becoming an increasingly frequent demand for anesthesiologists, since the growing use of video-assisted thoracoscopic surgery (VAST) at this age (1, 2).

The reduced dimensions of the airway in neonates and very small children limit the airway management to two approaches, endobronchial intubation with a single lumen tube (SLT) to the non-operating lung or a bronchial blocker (BB) placed in the operating lung (3–5). There are limitations reported from the use of the first technique in children up to 2 years old since the use of a larger or cuffed tube can cause tracheobronchial mucosa damage (6–9), and the use of a smaller tube can increase airflow resistance, create auto-peep, limit suctioning or be unable to provide adequate sealing (10, 11).

The lower tracheobronchial anatomy favors the insertion of the endobronchial tube to the right mainstem bronchus but with the risk of right upper lobe occlusion. On the other hand, the left main bronchus is smaller than the right, but the emergence of the upper left lobe is consistently more distal than the right side, reducing the risk of occlusion (5).

The use of a bronchial blocker is considered the technique of the choice for children under 6 years old (12). The Arndt Bronchial blocker has been the most popular device in the pediatric population due to its safety, high efficacy, and intuitive placement (12–16). In children under 2 years old, an extraluminal approach is preferred, since it allows the maintenance of driving pressure to ventilation, reducing the risk of a compromised oxygenation and ventilation during the device placement (2, 5, 17, 18). Although the complications of the airway instrumentalization with a bronchial blocker are rare, they can be potentially serious, including bronchial blocker entrapment (19), bronchial perforation and rupture (8, 20), and tip fracture (21, 22).

A flexible fiberoptic bronchoscope (FFB) is routinely recommended to guide the endobronchial intubation and the placement of the bronchial blocker to its final position (23–25).

The last decade is characterized by a significant dissemination of the use of ultrasound as a point-of-care methodology. In the pediatric population, with the arrival of small-size, high-frequency ultrasound probes a fast bed-sided examination of the airway can be done with efficiency and safety, reporting airway sonoanatomy and endotracheal tube position (26–28). The low mineral density and the incomplete fusion of the ossification centers of the sternum and ribs in neonates and small infants allow the use of ultrasound for mediastinum evaluation, including trachea and carina and provides a feasible window of observation (29–31).

Rabbits have been considered adequate models for experimental pediatric surgery and anesthesia, especially for neonatal and small children’s airway and ventilation research (32–35).

The purpose of this study is to evaluate the upper and lower airway sonoanatomy and describe an ultrasound-guided approach to selective bronchial intubation using an SLT and a BB in a pediatric animal model.

## Materials and Methods

### Ethical Approval

Ethical approval for this study was provided by EU Directive 2010/63/EU, under project authorization attributed by local ethics committee (*Subcomissão de Ética para as Ciências da Vida e da Saúde* – SECVS 004/2016, Chairperson Prof. Cecília Leão, on March 2016) and by the national authority for animal protection (*Direção Geral de Alimentação e Veterinária* - et al. DGAV 015296, Chairperson Dr. Fernando Bernardo, on June 2017). This manuscript adheres to the applicable EQUATOR guideline and was performed in accordance with ARRIVE guidelines (Supplementary Document 1).

### Animals and Anesthesia

Seven adult rabbits (*Oryctolagus cuniculus*), 4 females and 3 males were used (2242 g ± 151 g body weight average). Animals were anesthetized in their home pen with a subcutaneous administration of a combination of Ketamine (25 mg/kg; Ketamidor, Richter Pharma AG, Austria), Medetomidine (0.4 mg/kg; Sededorm, VetPharma Animal Health, Barcelona, Spain) and Buprenorphine (0.03 mg/kg; Bupaq, Richter Pharma AG, Austria) and transported to surgical laboratory. A peripheral venous access in the ear was obtained for fluid (0.9% saline, 2 ml/kg/h) and anesthesia administration. General anesthesia was maintained using continuous intravenous administration of propofol (4.2 mg/kg/h). Oxygenation was monitored using an oximeter and oxygen therapy was provided to an animal in spontaneous ventilation in order to maintain oxygenation above 95%. After tracheal intubation, animals were ventilated in pressure support mode. Anesthesia depth was increased for signs of inadequate anesthesia such as increased heart rate, small movements, or lacrimation.

Animals were positioned supine with heads extended and were shaved from the neck to the xiphoid process in a median plane and bilaterally in the thorax until the medial axillary line.

At the end of the experiment, with the animals still under deep anesthesia, euthanasia was performed by an intravenous administration of Pentobarbital (200 mg/kg; Euthasol, Le Vet Beheer B.V., Netherlands).

### Ultrasound Approach

The study included the animal upper and lower airway sonoanatomy evaluation and the real-time ultrasound scanning of the introduction of the SLT and the BB from glottis to right and left main bronchus, respectively.

The scanning protocol for the study included a cervical anterior approach with longitudinal and transverse planes, sliding the probe from the mentum to the suprasternal notch. The probe was then moved to the thoracic level, with an oblique parasternal, midclavicular plane from the suprasternal notch to the anterolateral face of the right hemithorax. The ultrasound penetration used a range between 2.0 and 3.0 cm depth. Ultrasound image acquisition was performed by an experienced anesthesiologist in human airway ultrasound, using a SonoSite Edge II (FUJIFILM SonoSite^®^, Inc., Bothell, WA, United States) with a linear multifrequency 6–13 MHz transducer probes (HFL38xi) from FUJIFILM SonoSite^®^, Inc. (Bothell, WA, United States).

### Procedure

After anesthesia and animal monitoring, a scanning protocol was done to identify and describe the animal airway sonoanatomy. Due to the small, dimensions of the glottis and its mucosa fragility, a flexible fibreoptic bronchoscope was used (Karl STORZ SE&Co, Tuttlingen, Germany, 5.2 mm × 65 cm) to guide tracheal intubation and the extraluminal bronchial blocker insertion, only at the level of the glottis (Supplementary Figure 1).

During the fibreoptic procedure, local anesthetic – lidocaine 1%, was sprayed in the airway, though an epidural catheter (Perifix^®^ Standard, Soft-tip; B.Braun Melsungen AG, Germany) in order to provide mucosa anesthesia and facilitate the procedure.

After SLT and BB introduction at the level of the glottis, two procedures were performed guided exclusively by ultrasound:

Right endobronchial intubation with a cuffless, reinforced, extra soft, single lumen tube, 2.5 mm (MallinckrodtTM, Covidien^®^, United States) with an internal and external diameter of 2.5 mm and 4.0 mm, respectively. The SLT was the same that provided tracheal intubation.

Left lung exclusion with a 5 Fr bronchial blocker (Uniblocker^®^ MBA, Fuji Systems Corporation) with an external diameter of 1.7 mm (length 400 mm, effective length 300 mm, cuff length 8 mm, max. volume 3 ml), used with an extraluminal approach.

Confirmation of the side of the instrumented bronchi was done in post-mortem evaluation.

## Results

### Sonoanatomy Study

**FIGURE 1:**
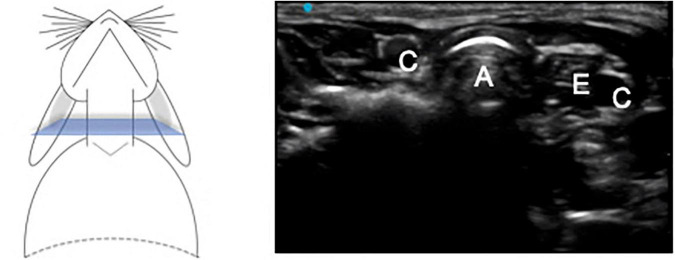
Transverse plane of the trachea at the middle cervical level. A – Trachea, C – Carotid artery, E – Esophagus.

**FIGURE 2:**
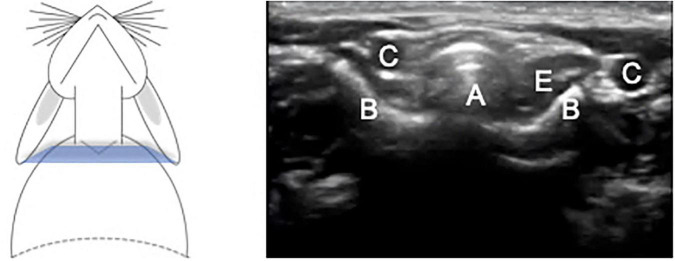
Transverse plane of the trachea at the caudal cervical level at the inlet of the thoracic cage. A – Trachea, B – Clavicle bone, C – Common Carotid artery, E – Esophagus.

**FIGURE 3:**
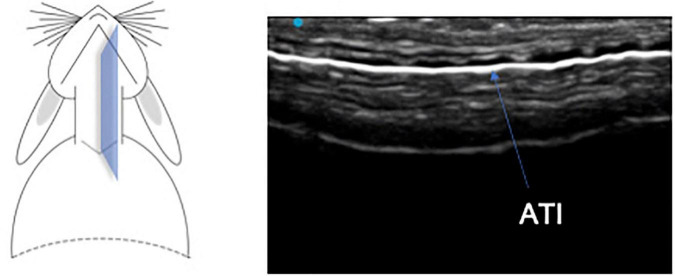
Longitudinal plane of cervical trachea. ATI, air-tissue interface.

The images from the cervical view in transverse and longitudinal planes are presented in Figures 1–3. The relationship between cervical trachea, esophagus and right and left common carotid artery was easy to identify.

**FIGURE 4:**
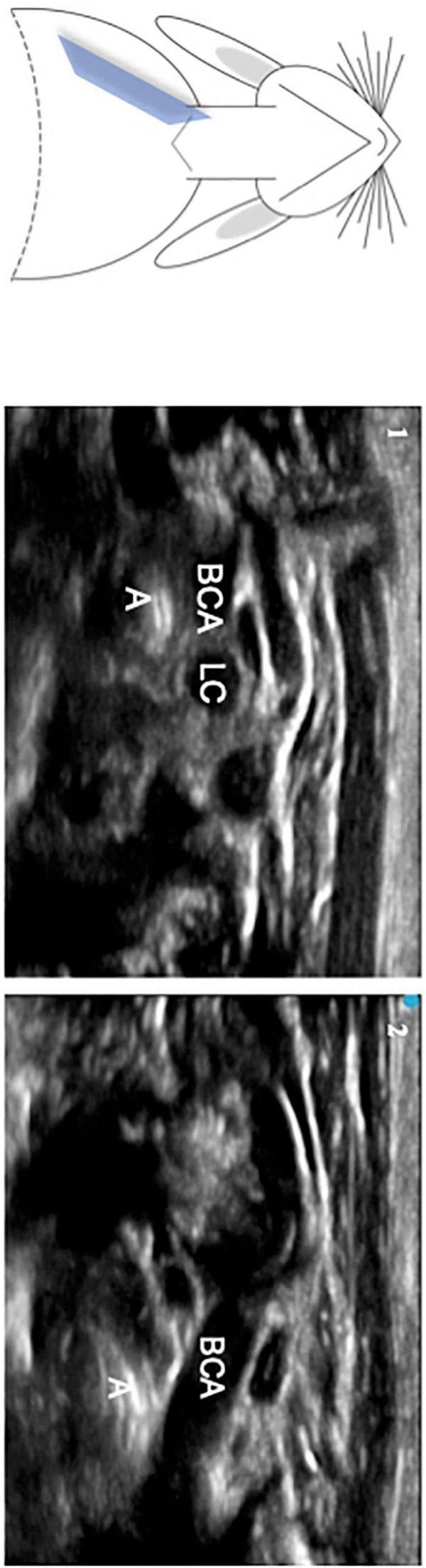
Oblique, parasternal, and midclavicular plane of the thoracic trachea. A – trachea, LC, Left common carotid artery, BCA, brachiocephalic or innominate artery. Part 1 – more cephalic view. Part 2 – more caudal view, at the level of the brachiocephalic artery.

In the right oblique, parasternal, and midclavicular plan, it was possible to visualize the intra-thoracic trachea, represented as two hyperechogenic lines, and its relationship with the aorta and innominate artery (Figure 4). The tracheal bifurcation was located at the level of the 3rd thoracic vertebra. During the scanning movement of the probe from intrathoracic to cervical level, it was possible to notice the trachea’s proximity to the aortic arch, innominate artery, right and left common carotid artery, and esophagus (Supplementary Video 1).

### Ultrasound Images of Single Lumen Tube and Bronchial Blocker Positioning

**FIGURE 5:**
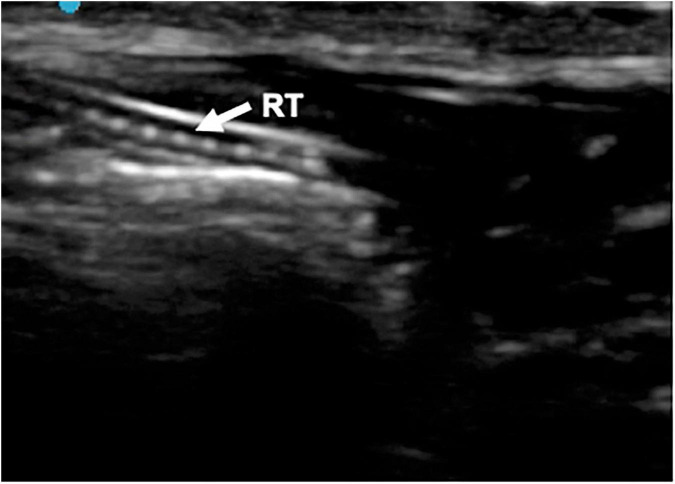
Longitudinal anterior plane of the neck. RT, reinforced tracheal tub.

It was possible to visualize in real-time, the progression of the SLT along the cervical trachea and its positioning in the right mainstem bronchus, with the probe moving from the cervical longitudinal to the oblique parasternal, midclavicular plane. Due to the artifact of the reinforced tube wire, a continuous ellipsis line was visible throughout the trachea extension (Figure 5).

**FIGURE 6:**
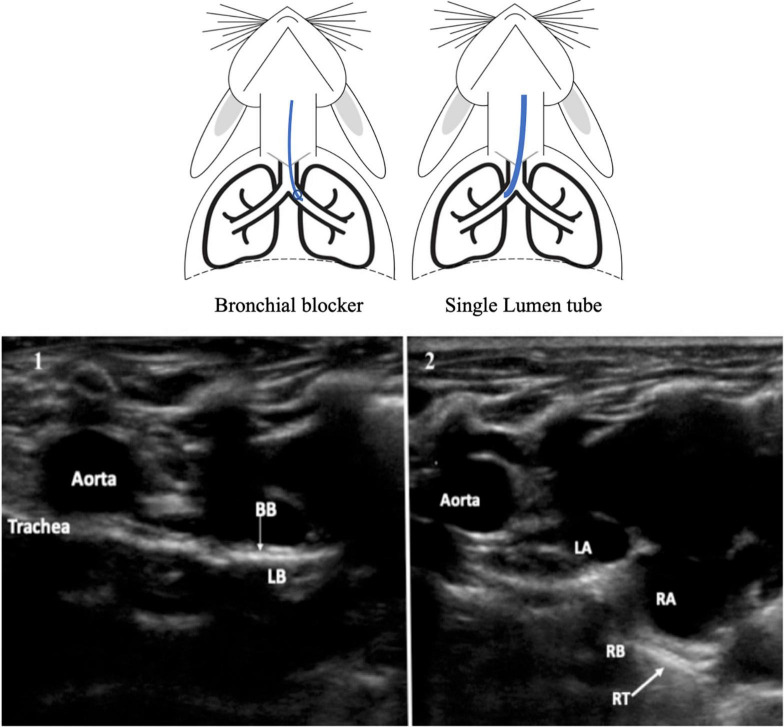
Oblique, parasternal, and midclavicular plane 1. Bronchial blocker in the left bronchus. BB, Bronchial blocker, LB, Left bronchus. 2. Reinforced tube in the right bronchus. RT, Reinforced tube, RB, Right Bronchus, LA, left atrium, RA, right atrium.

The progression of BB until the main left bronchus was visible with the ultrasound probe oriented in the same right oblique, parasternal, and midclavicular plane. The BB was seen as a dense line visible throughout the thoracic trachea and left bronchus (Figure 6 and Supplementary Video 2). Main right bronchus intubation was visualized with the probe in the same plane after a small probe tilts to the left. In this plane, the anatomic relationships between the right main bronchus and the right and left atrium were also identified.

In the first animal, with a fibreoptic view, a tracheal mucosa leak was identified during the first attempt at tracheal intubation. The animal was immediately euthanized and the experiment aborted. Considering that this animal was the smallest and the lightest of the sample, probably the size of the SLT (external diameter of 4 mm) was not appropriate for this animal. No other complications were reported.

All animals were euthanized after the experiments and the complementary anatomic study confirmed device positioning.

## Limitations

One potential limitation of the study is the use of a SLT only to the right side and the BB only to the left side positioning. However, in the work of Loewen et al. (36), small rabbits (weigh between 2.0 and 2.5 kg) had an average of tracheal ventral-dorsal diameter of 4.2 (min 3.75- max 4.75), with the left bronchus significatively smaller than the right. This animal findings are consistent with human studies. Hammer et al. (23) concluded by using computerized tomography that the left main bronchia is consistently smaller than the right and in children younger than 3 month its diameter can be smaller than the size of a 3.0 uncuffed endotracheal tube. Therefore, the positioning chosen for each device was adequate and intended to reduce the risk of injury.

The sample size of the study was small. Nevertheless, the study intended to be a proof-of-concept with evidence of feasibility and reproducibility of the results.

## Discussion

The study details the successful use of ultrasound to guide bronchial intubation with an SLT and exclusion with a BB, in the pediatric rabbit model (Supplementary Visual Abstract).

Rabbits are an adequate and useful animal model for studies related to airway management in pediatric ages such as (1) subglottic stenosis, since their larynx dimensions are similar in size; (2) diaphragmatic hernia repair; and (3) one-lung ventilation management (32–35, 37).

The anatomical characteristics of the narrow and deep oral cavity and the lining mucosa’s frailty make the management of the rabbit’s airway challenging. According to recent studies (38) optimizing the view of the glottis with fiberoptics can facilitate the intubation of very small lab animals. The use of fiberoptics in our study was limited to the intubation at the level of the glottis, with no further need for the FFB.

The concept of the study was to dispense the use of FFB for pediatric one-lung intubation or exclusion. In this population, the glottis view is well achieved through direct laryngoscopy or video-laryngoscopy, meaning that the glottis intubation is not an issue. However, published studies described the use of ultrasound as an alternative technique to guide real-time tracheal intubation in children (39), in patients with cervical trauma (40), or with an anticipated difficult airway (41, 42).

Hereby, studies with the exclusive use of the US to achieve glottis intubation and to monitor the correct positioning of the SLT or BB can bring promising results to clinical application.

We used a reinforced endotracheal tube to reduce the risk of occlusion since the small airway was also instrumented with a bronchial blocker. As a result, we identified an ultrasound image pattern represented by a continuous ellipsis line. This specific sign facilitated the identification of the tube in the trachea and right main bronchi, reducing the vision limitations from the air interference. The development of new models of BB with a metal wire coil embedded in the wall of the tube shaft can be transformative and allow an easier recognition of the device by the ultrasound.

In the sonoanatomy study, the relationships observed with ultrasound between cervical and intrathoracic trachea with the aorta, innominate artery and common carotid arteries are in complete accordance with the anatomical study previously done by Souza et al. (43). The bifurcation of the trachea was identified by ultrasound at the level of the 3rd intercostal space and was confirmed in the post-mortem study.

This study introduced in an animal model a safe, non-invasive and non-radiant technique that could independently guide SLT and BB positioning for one-lung ventilation purposes. The authors believe that this technique can be particularly useful for premature, neonate, and small children under 2 years old with a non-ossified thoracic wall. Nevertheless, this approach requires technical skills training to achieve competence, with its learning curve still to be defined.

## Conclusion

The study introduced the use of ultrasound to guide selective bronchial intubation and bronchial exclusion in a pediatric animal model, providing a new ultrasound application to airway management. These preclinical findings can drive new perspectives of research, training and clinical application, such as in neonatal and small children’s anesthesia.

## Data Availability Statement

The original contributions presented in the study are included in the article/Supplementary Material, further inquiries can be directed to the corresponding author.

## Ethics Statement

The animal study was reviewed and approved by the Chairperson Prof. Cecília Leão, SECVS 004/2016 Chairperson Dr. Fernando Bernardo, DGAV 015296.

## Author Contributions

SG, JC-P, and JP: conception or design of the study. SG, AM, and JP: acquisition, analysis, and interpretation of data. SG, AM, JP, PC, and JC-P: manuscript reviewing, final approval of the version to be published, and agreement to be accountable for all aspects of the study in ensuring that questions related to the accuracy or integrity of any part of the study are appropriately investigated and resolved. SG and AM: figures and movies. All the authors contributed substantially to the study.

## Conflict of Interest

The authors declare that the research was conducted in the absence of any commercial or financial relationships that could be construed as a potential conflict of interest.

## Publisher’s Note

All claims expressed in this article are solely those of the authors and do not necessarily represent those of their affiliated organizations, or those of the publisher, the editors and the reviewers. Any product that may be evaluated in this article, or claim that may be made by its manufacturer, is not guaranteed or endorsed by the publisher.

## Supplementary Material

Supplementary Figure 1 Flexible fibreoptic bronchoscope view with the glottis intubated with a single lumen tube and the extraluminal approach of the bronchial blocker.

Supplementary Video 1 Ultrasound video visualizing trachea’s proximity to the aortic arch, innominate artery, right and left carotid artery, and esophagus.

Supplementary Video 2 Ultrasound video with the identification of the bronchial blocker in the main left bronchus.

Supplementary Document 1 ARRIVE set document.

Supplementary Visual Abstract The study introduced an exclusive ultrasound-guided bronchial intubation with a reinforced single lumen tube and bronchial exclusion with a bronchial blocker technique on rabbit pediatric model. Further advanced experimental studies are needed for translation to pediatric anesthesia.
