# Supplementary figures and images for: Ultrasound-Guided Selective Bronchial Intubation: A Feasibility Study in Pediatric Animal Model
Source: Front Med (Lausanne). 2022 Jun 15;9:869771. doi: 10.3389/fmed.2022.869771 (PMC9240755; doi:10.3389/fmed.2022.869771)

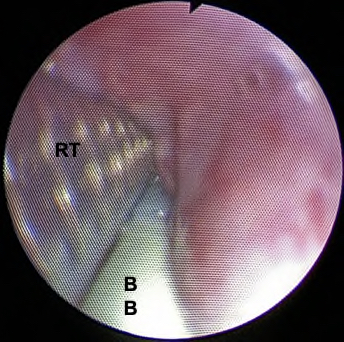

Supplement: Supplementary Figure 1 — Flexible fibreoptic bronchoscope view with the glottis intubated with a single lumen tube and the extraluminal approach of the bronchial blocker. [file Image_1.JPEG]
