# Supplementary material for: Ultrasound-Guided Selective Bronchial Intubation: A Feasibility Study in Pediatric Animal Model
Source: Front Med (Lausanne). 2022 Jun 15;9:869771. doi: 10.3389/fmed.2022.869771 (PMC9240755; doi:10.3389/fmed.2022.869771)
Supplement: Supplementary Document 1 — ARRIVE set document. [file Data_Sheet_1.pdf]

Compliance with ARRIVE Guidelines

Study design

This feasibility study aims to develop an animal model for exclusively ultrasound guide endobronchial intubation and bronchial exclusion for paediatric one lung ventilation. It does not have animals allocated in different groups. The animals were clearly identified for the study.

Sample Size

No power calculation was done. Authors decided to include only seven animals since this is a study to develop an animal model for a procedure intervention. As in previous preclinical studies the number of animals was inferior to ten.

Inclusion and exclusion criteria

Not applicable to the study design

Randomization

No randomization was done, because it’s not applicable to the study design.

Blinding

Not applicable to the study

Outcome measures

The primary outcome of the study was do develop a paediatric animal model for exclusively ultrasound guided one lung ventilation through bronchial intubation or bronchial exclusion.

Statistical methods

No statistical methods were done, since it is a preclinical animal model for a procedure.

Experimental animals

The species, sex, age and weight were specified. Seven adult rabbits (*Oryctolagus cuniculus*), 4 females and 3 males were used (2242g±151g body weight average).

Experimental procedures

Animals were anesthetized in their home pen with a subcutaneous administration of a combination of Ketamine, Medetomidine and Buprenorphine and transported to surgical laboratory. A peripheral venous access in the ear was obtained for fluid and anaesthesia maintenance with Propofol. Oxygenation was monitored using an oximeter and oxygen therapy was provided to animal in spontaneous ventilation in order to maintain oxygenation above 95%.

Animals were shaved from the neck to the xiphoid process in a median plane and bilaterally in the thorax until the medial axillary line.

Intubation with a fibreoptic bronchoscope was done as well as the introduction of a bronchial blocker with an extraluminal approach. Ultrasound imaging acquisition was done in cervical and thoracic area. Ultrasound guided a single lumen tube intubation of the right bronchus and a bronchial blocker positioning in the left bronchus.

At the end of the experience, with the animals still under anaesthesia, euthanasia was performed by an intravenous administration of Pentobarbital. Confirmation of the side of the instrumented bronchi was done in post-mortem evaluation.

Results

Descriptive statistics nor effect size or confidence interval were not provided, since were not applicable to the type of data collected. An ultrasound scanning was successfully done with the identification of the airway sonoanatomy since cervical trachea to carina. Anatomic relationships between intra-thoracic trachea and aorta, innominate and carotid arteries and oesophagus were observed. The management of the tube and the bronchial blocker were completely identified in real-time with ultrasound. In conclusion, this study showed that it is possible to guide exclusively by ultrasound the endobronchial intubation and bronchial exclusion in this animal model.

Authors believe that this paper can open to new directions for research and clinical application, namely neonatal and paediatric anaesthesia.
