# Supplementary material for: Ultrasound-Guided Selective Bronchial Intubation: A Feasibility Study in Pediatric Animal Model
Source: Front Med (Lausanne). 2022 Jun 15;9:869771. doi: 10.3389/fmed.2022.869771 (PMC9240755; doi:10.3389/fmed.2022.869771)
Supplement: Supplementary Visual Abstract — The study introduced an exclusive ultrasound-guided bronchial intubation with a reinforced single lumen tube and bronchial exclusion with a bronchial blocker technique on rabbit pediatric model. Further advanced experimental studies are needed for translation to pediatric anesthesia. [file Data_Sheet_2.pdf]

# Ultrasound-guided selective bronchial intubation - a feasibilty study in pediatric animal model

## Prospective experimental study

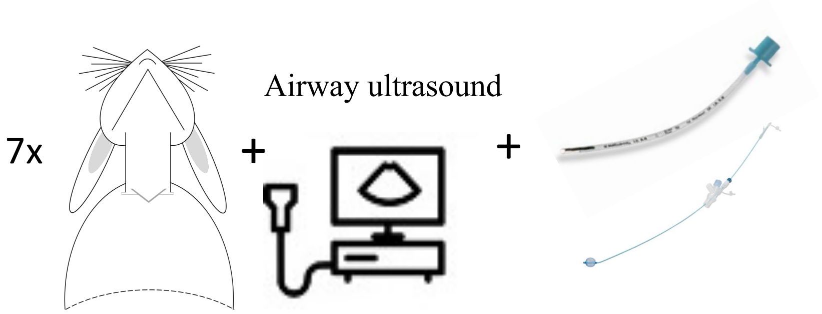


Ultrasound probe orientation

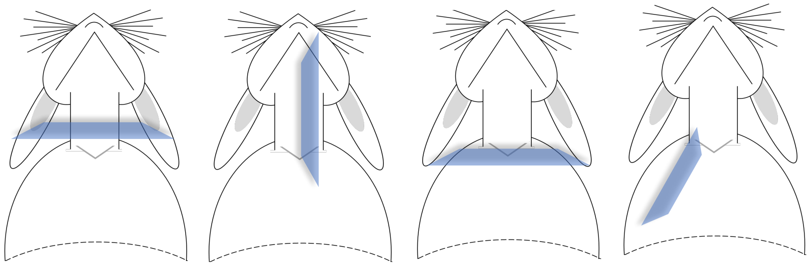

## Results

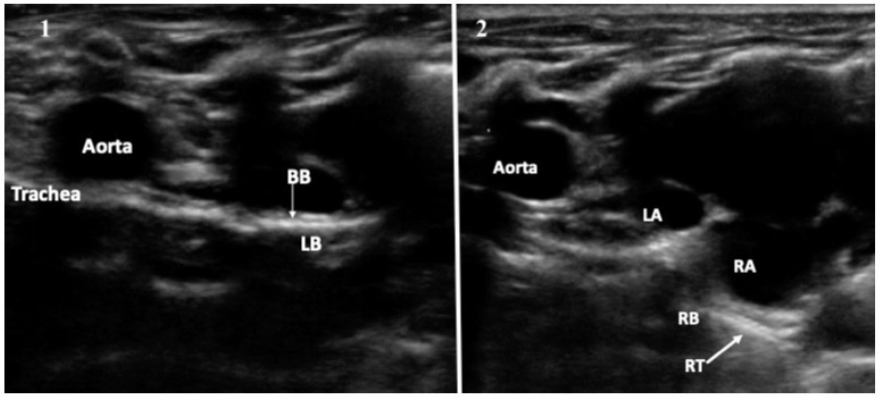


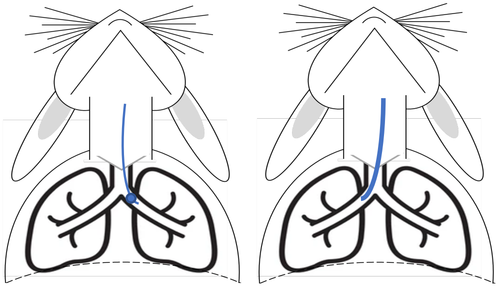

Bronchial blocker   Single Lumen tube

In animal model, ultrasound was able to exclusively guide the positining of a bronchial blocker and a single lumen tube in order to provide one-lung ventilation.

**Implications**

More advanced experimental studies are needed since this technique has the potential for translation to pediatric anesthesia
